# Effect and safety of extracorporeal shockwave therapy for postherpetic neuralgia: A randomized single-blind clinical study

**DOI:** 10.3389/fneur.2022.948024

**Published:** 2022-09-26

**Authors:** Lu Chen, Ailing Qing, Tao Zhu, Pingliang Yang, Ling Ye

**Affiliations:** ^1^Department of Pain Management, West China Hospital, Sichuan University, Chengdu, China; ^2^Department of Anesthesiology, West China School of Public Health and West China Fourth Hospital, Sichuan University, Chengdu, China; ^3^Department of Anesthesiology, West China Hospital of Sichuan University, Chengdu, China; ^4^Department of Anesthesiology, The First Affiliated Hospital of Chengdu Medical College, Xindu, China

**Keywords:** extracorporeal shockwave therapy, postherpetic neuralgia, neuropathic pain, chronic pain, quality of life

## Abstract

**Objective:**

To evaluate the efficacy and safety of extracorporeal shockwave therapy (ESWT) for postherpetic neuralgia.

**Design:**

Randomized single-blind clinical study.

**Patients:**

Patients with postherpetic neuralgia.

**Methods:**

Patients were randomly divided into the control group and the ESWT group. The control group received conventional treatment while the ESWT group received conventional treatment and ESWT. The primary outcome is pain degree as assessed by the numeric rating scale (NRS), and secondary outcomes include brief pain inventory (BPI), Self-rating Anxiety Scale (SAS), Self-rating Depression Scale (SDS), and Pittsburgh Sleep Quality Index (PSQI). Data were collected at baseline and at weeks 1, 4, and 12. Linear mixed-effects models were applied to repeated measurement data.

**Results:**

The scores on the NRS, BPI, SAS, SDS, and PSQI decreased over time in both groups. The NRS and SDS scores of the ESWT group were statistically lower than the control group. There was no time × group interaction in the mixed model analysis. Baseline age was correlated with NRS scores and BPI scores, and invasive treatment was related to PSQI scores, with no interaction effect for baseline confounders observed. No adverse events were observed during the process of this trial.

**Conclusion:**

Extracorporeal shockwave therapy combined with conventional treatment could relieve pain and improve the psychological state in patients with postherpetic neuralgia without serious adverse effects.

## Introduction

Postherpetic neuralgia (PHN) is defined as persistent pain lasting more than 3 months after the onset of shingles (1), which is the most common complication of herpes zoster occurring in 20–30% of patients (2–5). As a type of neuropathic pain, PHN is related to peripheral-nerve damage and characterized by burning, itching, lightning, and sharp pain, some with allodynia or hyperalgesia, which seriously affect patients' life quality, sleep quality, and mental state (3). The current common treatment for PHN includes medication and interventional therapy. First-line medication therapy includes pregabalin, gabapentin, and tricyclic antidepressants, while interventional therapy includes subcutaneous injection, electrical nerve stimulation, nerve block, pulsed radiofrequency, and spinal cord stimulation (6). Despite multiple treatments, there are still adverse effects, such as nausea, vomiting, and constipation caused by medication, as well as infection, bleeding, and nerve injury related to interventional therapy. Therefore, it is necessary to find an effective and non-invasive treatment for PHN.

A shockwave is a type of transient pressure fluctuation generated by electromagnetic, electrohydraulic, or piezoelectric devices (7). As an effective and safe treatment, extracorporeal shockwave therapy (ESWT) is widely used in urinary disease and musculoskeletal disorders (8–10). The main biological mechanism of ESWT includes wound healing, tissue regeneration, bone remodeling, angiogenesis, and anti-inflammation (11). In recent studies, ESWT has been reported to improve neuropathic pain, such as Morton's neuroma, primary trigeminal neuralgia, and diabetic neuropathy (12–15). In addition, a pilot study that includes 13 patients suggested that ESWT could significantly reduce symptoms of PHN (16). However, a randomized controlled trial with a larger sample size and longer follow-up is lacking. The objective of this study is to evaluate the effect and safety of ESWT on patients with PHN in short and middle term.

## Materials and methods

### Design

This study design is a single-center, single-blind, randomized controlled trial, which was approved by the Ethics Committee of West China Hospital, Sichuan University, Chengdu, China (No. 2019[814], date of approval: 30 December 2019) and registered at ChiCTR.org.cn (Identifier: ChiCTR1900025828, date of registration: 10 September 2019). The initial version of the protocol was published (17). Consolidated Standards of Reporting Trials (CONSORT) were followed.

### Participants

Patients were recruited from September 2019 to September 2020 in the pain department of West China Hospital. After being informed of the procedures and possible complications of the study, they decided whether to participate in this study. Then, more detailed information was collected to assess the eligibility. The inclusion criteria included adults diagnosed with PHN according to the Consensus of Chinese experts on PHN (18); had an NRS score ≥4 points; had described symptoms objectively; had not received ESWT previously; and had not participated in other clinical trials within 3 months. The exclusion criteria included patients who had a history of allergy to coupling agent; tumor; liver or kidney dysfunction; thrombosis or abnormal coagulation; with a cardiac pacemaker; infectious; pregnant; fracture or severe osteoporosis; and mental disorders.

### Randomization and blinding

After signing the informed consent, the participants were allocated into the control group or the ESWT group (1:1). In this process, a researcher was in charge of preparing the sealed opaque envelopes, which contained random numbers generated by EXCEL table. Another researcher was responsible for assigning the envelope to participants randomly, and then a shockwave therapist decided whether to perform shockwave therapy according to the random numbers in envelopes. The assessors and statisticians were blinded to randomization and did not participate in the treatment.

### Treatment and outcomes

The control group received conventional treatment, such as medication and invasive interventional therapy, while the ESWT group received conventional treatment and extracorporeal shockwave therapy. Conventional treatment remained stable during the study period in patients receiving ESWT. The detailed therapeutic schedule is shown in Table 1. The schedule of drugs was adjusted based on the patients' symptoms, while the selection of invasive interventional therapy depended on the painful area and course of disease.

**Table 1:** Therapeutic schedule.

	**Therapy**		**Schedule**
Conventional treatment	Medication	Gabapentin	0.3 g qd on day 1
			0.3 g bid on day 2
			0.3 g tid on day 3 and maintained
		Pregabalin	75 mg bid
		Oxycodone and acetaminophen	0.5 tablet tid
		Mecobalamin	0.5 mg tid
	Invasive interventional therapy	Epidural nerve block	2 ml 2% Lidocaine
			1 ml compound Betamethasone
			2 ml Mecobalamin for injection
			5 ml normal saline
		Radiofrequency modulation	42°C 65 V, 15 min
		Radiofrequency thermocoagulation	65°C, 30 s; 70°C, 30 s
			75°C, 1 min; 80°C, 2 min; 85°C, 2 min
Extracorporeal shockwave therapy			10 Hz; 1–4 bar; 4000–7000 pulses performed every 3–5 days
			3–5 sessions consist a course

qd, once a day; bid, two times a day; tid, three times a day.

**Figure 1:**
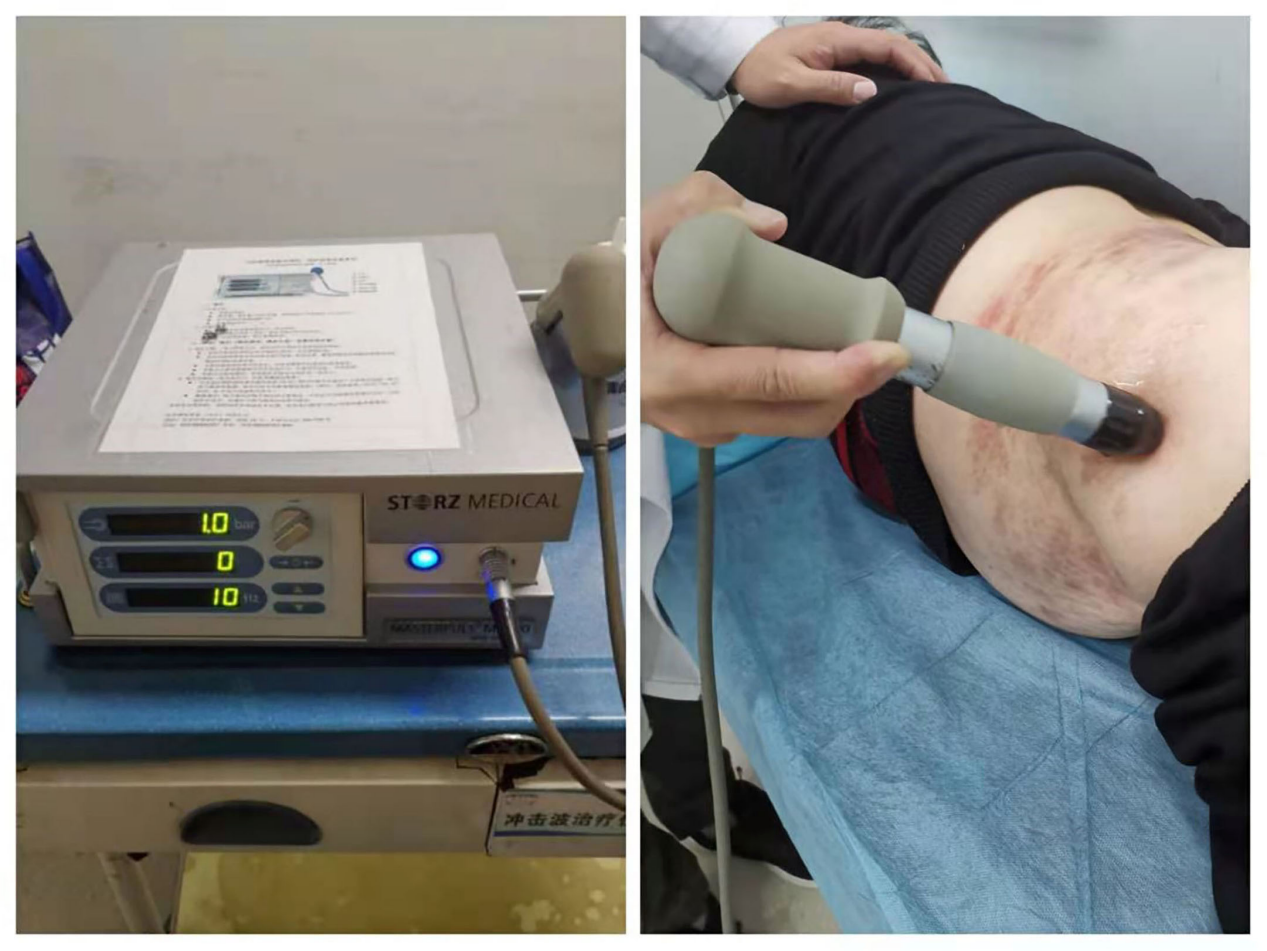
Extracorporeal shockwave therapy (ESWT) for postherpetic neuralgia (PHN). The patient was treated by a radial extracorporeal shockwave generator (MASTERPULS MP100; Storz Medical AG, Switzerland) in a lateral position.

Extracorporeal shockwave therapy was performed by a skilled therapist with a radial extracorporeal shockwave generator (MASTERPULS MP100; Storz Medical AG, Switzerland). As shown in Figure 1, the patients could be in different positions (prone, lateral, or seated position) depending on the location of skin lesions. After applying the coupling agent to the skin, a R15 probe (radius of 15 mm) was moved along the nerve. The energy could gradually increase according to the patients' reaction. The primary outcome is pain intensity assessed by the numeric rating scale (NRS), and secondary outcomes include quality of life assessed by brief pain inventory (BPI), psychological state assessed by the Self-rating Anxiety Scale (SAS) and the self-rating Depression Scale (SDS), and sleep quality assessed by the Pittsburgh Sleep Quality Index (PSQI). Assessors collected data by telephone interviews at baseline and at weeks 1, 4, and 12. Adverse reactions related to ESWT were recorded to evaluate the safety.

### Statistical analysis

The sample size was estimated with the superiority test (α = 0.05 and β = 0.2). According to a previous study (19), we calculated that 76 participants were required. Demographic and baseline characteristics included age, sex, BMI, nerve segments of PHN, PHN duration, medication, invasive therapy, and per capital invasive therapy course. In the analysis of baseline data, quantitative data with normal distribution were presented as mean ± standard deviation (SD) and analyzed by the independent-samples *T*-test, while data that did not conform with normal distribution were presented as median (the upper and lower quartiles) and analyzed by the Mann–Whitney *U*-test. Categorical data were presented as frequency (percentage) and compared by the χ^2^ test. In the analysis of repeated measurement data, intention-to-treat (ITT) analysis was conducted with the missing data replaced by using the last observation carried forward (LOCF) imputation method. Linear mixed-effects models were applied to longitudinal data, and restricted maximum likelihood (REML) with an unstructured covariance matrix was used. In the 2 groups (ESWT vs. control), 4 time points (baseline, weeks 1, 4, and 12), and the time × group interaction were considered as fixed effects, while baseline confounders, such as age, sex, body mass index (BMI), invasive treatment, and PHN nerve segments as covariates. IBM SPSS Statistics 26 was used for statistical analysis. All tests were two-sided, with *p* < 0.05 indicating statistical significance.

## Results

### Patient flow

**Figure 2:**
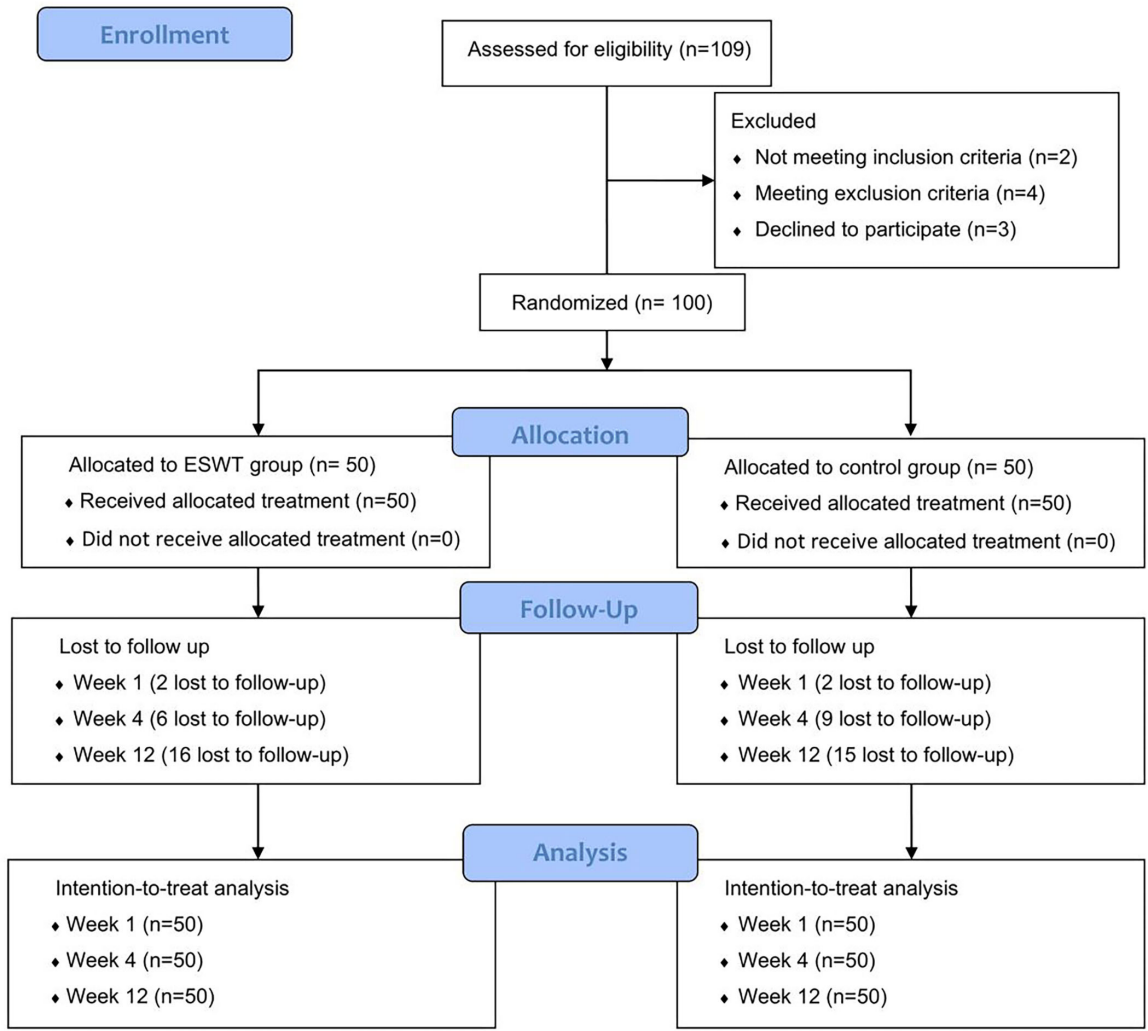
Consort flow diagram. A total of 100 participates were randomized into the control group and the ESWT group, who were followed up at weeks 1, 4, and 12. All participates were included in the intention-to treat analysis.

In this study, 109 patients were recruited and assessed for eligibility and a total of 100 patients were included. After being randomized in a 1:1 ratio to the two groups, they received their allocated treatment. At week 1, 4 patients were lost to follow-up, 15 patients were lost to follow-up at week 4, and 31 patients were lost to follow-up at week 12. Finally, 69 patients finished 12-week follow-up. The data of all participates were included in the ITT analysis (Figure 2).

### Demographic and baseline characteristics

There were no significant differences between the ESWT group and the control group in age, gender, body mass index (BMI), PHN localization, PHN duration, medication therapy, and invasive therapy, suggesting that the baselines were comparable (Table 2).

**Table 2:** Demographic and baseline characteristics of participates.

**Variable**	**All (*****n*** = **100)**			**Finished follow-up (*****n*** = **69)**			**Lost follow-up (*****n*** = **31)**			**All (*****n*** = **100)**		
	**ESWT (*n* = 50)**	**Control (*n* = 50)**	***P***	**ESWT (*n* = 34)**	**Control (*n* = 35)**	***P***	**ESWT (*n* = 16)**	**Control (*n* = 15)**	***P***	**Finished (*n* = 69)**	**Lost (*n* = 31)**	***P***
Age, years	67.9 ± 10.8	67.4 ± 11.2	0.84	64.15 ± 8.89	65.71 ± 11.96	0.540	75.75 ± 10.35	71.33 ± 8.24	0.201	64.94 ± 10.51	73.61 ± 9.50	**< 0.001**
Male sex, *n* (%)	23 (46%)	23 (46%)	1	20 (58.8%)	15 (42.86%)	0.185	3 (18.8%)	8 (53.3%)	**0.044**	35 (50.7%)	11 (35.4%)	0.157
BMI (kg/m^2^)	23.16 ± 3.12	23.40 ± 3.36	0.705	24.10 ± 2.92	23.43 ± 3.61	0.398	21.15 ± 2.59	23.34 ± 2.79	**0.031**	23.76 ± 3.28	22.21 ± 2.87	**0.026**
PHN segment, *n* (%)												
Cervical-facial segment	12 (24%)	10 (20%)	0.59	8 (23.5%)	6 (17.1%)	0.248	4 (25%)	4 (26.7%)	0.562	14 (20.3%)	8 (25.8%)	0.644
Thoracic segment	34 (68%)	38 (76%)		22 (64.7%)	28 (80%)		12 (75%)	10 (66.6%)		50 (72.5%)	22 (71.0%)	
Sacral-lumbar segment	4 (8%)	2 (4%)		4 (11.8%)	1 (2.9%)		0 (0%)	1 (6.7%)		5 (7.2%)	1 (3.2%)	
PHN duration, months	2 (1–3.25)	2 (1–6)	0.2	2 (1–3.25)	2 (1–6)	0.546	2 (1–5.25)	3 (2–7)	0.175	2 (1–5)	2 (1–6)	0.269
Medication									/			
Gabapentin g/d	0.9 (0.9–1.125)	0.9 (0.9–0.9)	0.981	0.9 (0.9–1.125)	0.9 (0.9–1.125)	0.886	/	0.9 (0.9–0.9)	0.110	0.9 (0.9–0.9)	0.9 (0.9–0.9)	0.728
Pregabalin mg/d	150 (150–225)	150 (150–225)	0.856	150 (150–225)	150 (225–300)	0.290	150 (150–225)	150 (150–150)		150 (150–225)	150 (150–150)	0.169
Invasive therapy, *n* (%)												
Epidural nerve block	3 (6%)	6 (12%)	0.657	3 (8.8%)	5 (14.3%)	0.773	0 (0%)	1 (6.7%)	0.151	8 (11.6%)	1 (3.2%)	0.576
Radiofrequency modulation	25 (50%)	22 (44%)		16 (47.1%)	15 (42.9%)		9 (56.3%)	7 (46.7%)		31 (44.9%)	16 (51.6%)	
Radiofrequency thermocoagulation	14 (28%)	16 (32%)		9 (26.5%)	11 (31.4%)		5 (31.2%)	5 (33.3%)		20 (29.0%)	10 (32.3%)	
Combined therapy	8 (16%)	6 (12%)		6 (17.6%)	4 (11.4%)		2 (12.5%)	2 (13.3%)		10 (14.5%)	4 (12.9%)	
Per capital invasive therapy course, times	2 (1~3)	2 (2~3)	0.61	2 (2–3)	2 (2–3)	0.630	3 (2–3.75)	2 (2–3)	0.748	2 (2–3)	2 (2–3)	0.279

PHN, postherpetic neuralgia; BMI, body mass index; ESWT, extracorporeal shockwave therapy.

A total of 31 participants were lost to follow-up because nobody answered the phone. The demographic and baseline characteristics of lost patients indicated that they were significantly older (*p* < 0.001) with lower BMI (*p* = 0.026) than patients who finished follow-up, while there was no significant difference in sex, PHN nerve segments, PHN duration, medication therapy, invasive therapy, and per capital invasive therapy course (Table 2).

Of the 31 patients who lost to the follow-up, 16 patients were in the treatment group while 15 patients were in the control group. There was a statistical difference in sex (*p* = 0.044) and BMI (*p* = 0.031) between the two groups. In 69 patients who finished 12-week follow-up, there were no significant difference in demographic and baseline characteristics between the ESWT group and the control group (Table 2).

### Primary outcome

**Figure 3:**
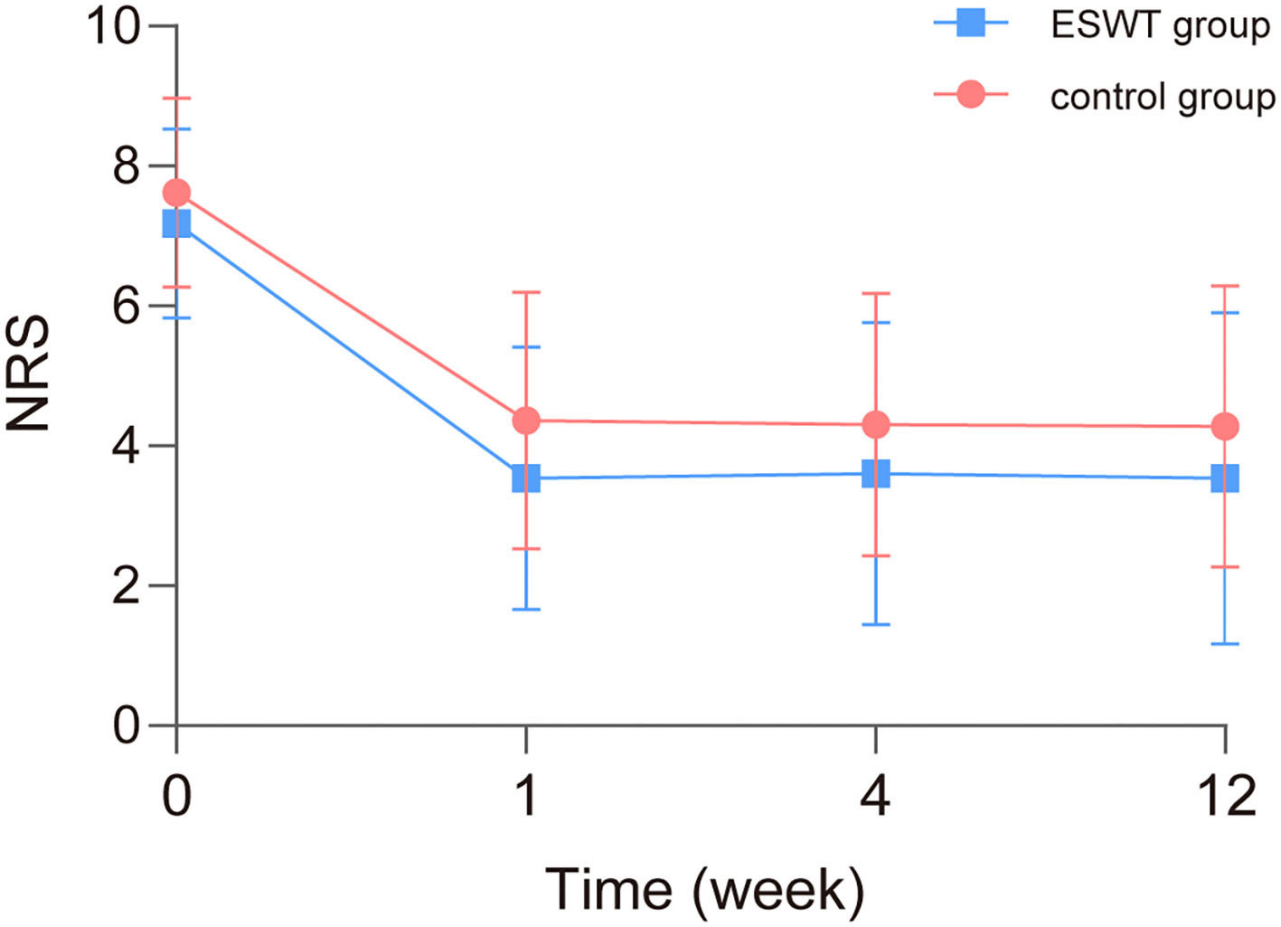
Baseline and post-treatment numeric rating scale (NRS) scores. The NRS scores in the ESWT group and control group decreased over time similarly. NRS scores of the ESWT group were statistically lower than the control group.

The scores and trends of baseline and post-treatment NRS scores are shown in Table 3 and Figure 3. The analysis of interaction effects for baseline confounders suggested that age was correlated with the NRS score but there was no interaction effect. A time × group interaction term was added in the mixed model, which indicated that the NRS scores in the ESWT group and the control group followed similar trends over time (*p* > 0.05), then the model was refitted without the time × group interaction term (Table 4). The final model showed that NRS scores was statistically associated with group (*p* = 0.027) and time (*p* < 0.001). The NRS scores decreased over time, and the NRS scores of the ESWT group were statistically lower than the control group.

**Table 3:** Baseline and post-treatment outcome scores.

	**NRS**	**BPI**	**SAS**	**SDS**	**PSQI**
**Baseline**					
Control	7.62 ± 1.35	37.88 ± 9.18	40.48 ± 6.64	41.96 ± 7.76	15.76 ± 4.80
ESWT	7.18 ± 1.35	41.36 ± 12.35	42.52 ± 6.76	38.76 ± 7.41	18.04 ± 5.71
**Week 1**					
Control	4.36 ± 1.84	25.14 ± 11.30	36.42 ± 7.16	35.96 ± 8.16	14.62 ± 5.15
ESWT	3.54 ± 1.87	20.62 ± 13.22	31.00 ± 4.06	29.06 ± 3.99	9.82 ± 5.45
**Week 4**					
Control	4.30 ± 1.87	24.56 ± 11.41	35.98 ± 7.03	34.88 ± 7.49	14.20 ± 5.03
ESWT	3.60 ± 2.16	18.90 ± 13.29	30.28 ± 4.45	28.52 ± 3.61	9.62 ± 6.07
**Week 12**					
Control	4.28 ± 2.01	24.04 ± 12.34	35.62 ± 7.49	34.04 ± 7.67	14.40 ± 5.57
ESWT	3.54 ± 2.36	18.28 ± 13.50	29.62 ± 4.32	28.24 ± 3.60	9.50 ± 6.03

ESWT, extracorporeal shockwave therapy; NRS, numeric rating scales; BPI, brief pain inventory; SAS, self-rating anxiety scale; SDS, self-rating depression scale; PSQI, Pittsburgh sleep quality index.

**Table 4:** Longitudinal change in scores for intervention related to the control group from baseline through 12 weeks.

**Parameter**	**NRS**			**BPI**			**SAS**			**SDS**			**PSQI**		
	**β(SE)**	***t***	***P***	**β(SE)**	***t***	***P***	**β(SE)**	***t***	***P***	**β(SE)**	***t***	***P***	**β(SE)**	***t***	***P***
Group															
control	Ref			Ref			Ref			Ref			Ref		
ESWT	−0.589 (0.262)	−2.245	**0.027**	−0.057 (2.002)	−0.028	0.977	−0.485 (1.55)	−0.313	0.755	−5.07 (1.632)	−3.107	**0.003**	−1.153 (0.933)	−1.236	0.220
Time	−0.268 (0.056)	−4.825	**< 0.001**	−2.111 (0.317)	−6.653	**< 0.001**	−2.542 (0.599)	−4.247	**< 0.001**	−1.528 (0.454)	−3.366	**0.001**	−0.632 (0.14)	−4.514	**< 0.001**
Sex															
Female	Ref			Ref			Ref			Ref			Ref		
Male	−0.071 (0.268)	−0.265	0.792	0.198 (2.041)	0.097	0.923	−0.102 (1.58)	−0.065	0.948	−0.595 (1.664)	−0.358	0.722	−1.545 (0.951)	−1.624	0.108
PHN nerve segment															
Lumbar segment	Ref			Ref			Ref			Ref			Ref		
Sacral segment	−0.976 (1.086)	−0.898	0.371	2.043 (8.286)	0.247	0.806	7.338 (6.413)	1.144	0.256	−4.484 (6.755)	−0.664	0.508	2.297 (3.862)	0.595	0.554
Cervical segment	−0.21 (0.805)	−0.262	0.794	−3.507 (6.138)	−0.571	0.569	−1.109 (4.751)	−0.233	0.816	−5.756 (5.004)	−1.150	0.253	−2.354 (2.861)	−0.823	0.413
Thoracic segment	−0.568 (0.784)	−0.725	0.470	−2.687 (5.979)	−0.449	0.654	−0.599 (4.627)	−0.129	0.897	−6.648 (4.874)	−1.364	0.176	−2.936 (2.786)	−1.054	0.295
Age	0.032 (0.013)	2.553	**0.012**	0.252 (0.097)	2.600	**0.011**	−0.026 (0.075)	−0.352	0.726	−0.064 (0.079)	−0.804	0.423	0.059 (0.045)	1.313	0.192
BMI	0.009 (0.042)	0.218	0.828	−0.091 (0.319)	−0.284	0.777	−0.046 (0.247)	−0.187	0.852	0.406 (0.26)	1.562	0.122	−0.044 (0.149)	−0.296	0.768
Invasive treatment	0.08 (0.139)	0.572	0.569	0.034 (1.061)	0.032	0.974	−0.564 (0.822)	−0.686	0.494	0.065 (0.865)	0.075	0.941	1.309 (0.495)	2.647	**0.010**

PHN, postherpetic neuralgia; BMI, body mass index; ESWT, extracorporeal shockwave therapy; NRS, numeric rating scale; BPI, brief pain inventory; SAS, self-rating anxiety scale; SDS, self-rating depression scale; PSQI, pittsburgh sleep quality index.

### Secondary outcome

**Figure 4:**
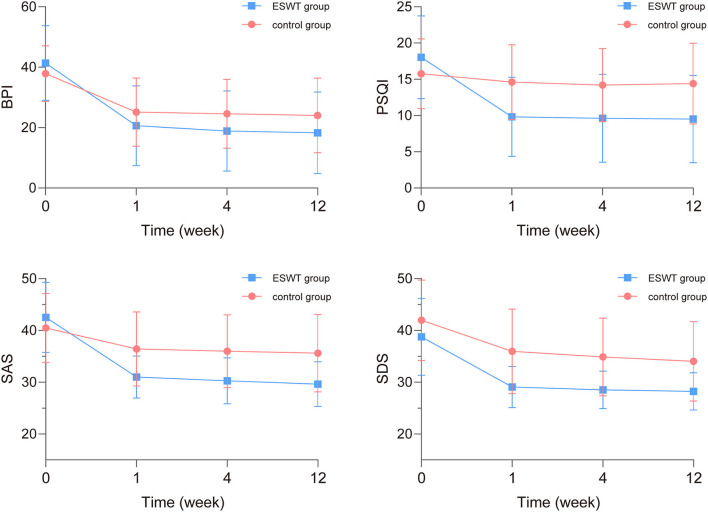
The baseline and post-treatment brief pain inventory (BPI), self-rating anxiety scale (SAS), self-rating depression scale (SDS), and pittsburgh sleep quality index (PSQI) scores. The BPI, SAS, SDS, and PSQI scores in the ESWT group and the control group decreased over time while SDS scores in the ESWT group were statistically lower than the control group.

The scores and trends of baseline and post-treatment BPI, SAS, SDS, and PSQI scores are shown in Table 3 and Figure 4. In the analysis of interaction effects for baseline confounders, age was associated with BPI scores while invasive treatment was related to PSQI scores. There was no interaction effect of confounders in this study.

The analysis of time × group interaction showed that the BPI, SAS, SDS, and PSQI scores changed similarly in both groups over time (*p* > 0.05). After readjusting the model without the time × group interaction term, the final model showed that SDS scores were statistically associated with group (*p* = 0.003) and the BPI, SAS, SDS, and PSQI scores were statistically associated with time (*p* < 0.001; Table 4). The BPI, SAS, SDS, and PSQI scores decreased over time while SDS scores in the ESWT group were statistically lower than the control group.

### Adverse reactions

There were no patient complaints about adverse reactions related to ESWT in either group through the follow-up period, such as skin swelling, allergy, fever, pain aggravation, paresthesia, tissue edema, and other adverse effects.

## Discussion

To our knowledge, ectopic activity, central sensitization, and inflammatory mediators might contribute to the pathophysiological mechanisms of neuropathic pain (20, 21). As a common type of neuropathic pain, PHN is related to varicella zoster virus, which remains dormant in the nerve after the primary infection and causes local rash and skin pain after reaction because of weakened immunity (22). The injury of the nerve could lead to the activation and migration of macrophages, release of proinflammatory cytokines, such as tumor necrosis factor α (TNF-α), which might contribute to hyperalgesia (20). In a previous study, ESWT may reduce the plasma levels of TNF-α and substance *P* (23). Therefore, the researchers designed this study to confirm the efficacy of ESWT for PHN based on the immune regulation and analgesic effect of ESWT.

In terms of demographic and baseline characteristics, the mean age of the patients was 67.9 (10.8) years in the ESWT group and 67.4 (11.2) years in the control group. Similarly, a previous study suggested that the risk of PHN rose sharply between 50 and 79 years (24). Advanced age was suggested to be a risk factor associated with PHN (4), which may be related to decreased immunity and weakened self-repair ability of the elderly, and the effect of age may differ in gender (25). Furthermore, female patients (54%) were more than male patients (46%) in our study and the risk of PHN was suggested higher in female patients (24). However, another study suggested that there was no significant difference in gender in the development of PHN in patients with Herpes zoster (HZ) (26). The correlation between gender and PHN was still controversial and more evidence is needed. In addition, the most commonly PHN localization was thoracic in our result, which was similar to another study (25).

The reason for patients' being lost to follow-up was that nobody answered the phone. The demographic and baseline characteristics of lost patients indicated that they were significantly older (73.61 years) than patients who finished follow-up (64.94 years). Patients who did not answer the phone might be related to the low smartphone adoption and unskilled use of smartphone in Chinese elderly people (27).

Considering that pain is an emotional experience and sense is the gold standard of pain (28), the researchers selected the NRS score as the primary outcome. In addition to pain, psychological disorders, such as anxiety and depression are common symptoms of patients with PHN, which could worsen pain, cause disability, and effect quality of life and sleep (29, 30). As a result, life quality, psychological state, and sleep quality were assessed as secondary outcomes to evaluate the efficacy of ESWT. The result indicated that there was no time × group interaction in the NRS, BPI, SAS, SDS, and PSQI scores, which indicated that these outcomes in the ESWT group and the control group followed similar trends over time. The NRS, BPI, SAS, SDS, and PSQI scores were statistically decreased with time in both groups. The NRS scores of the ESWT group were statistically lower than the control group, which may be related to lower sensory nerve conduction velocities, which result in altered peripheral pain perception after ESWT (31). In addition, the molecular neurobiology of chronic pain-induced depression contains genetic modifications, epigenetic modifications, transcription factors, and neurotransmitters (32). The reduced SDS scores and improved depression state in the ESWT group compared with the control group may be associated with analgesic effects and neuroinflammatory alterations caused by ESWT, such as TNF-α, which could be explored in further study. Although the BPI, SAS, PSQI scores in the ESWT group were lower than the control group, the difference was not significant, which might because both groups improved well over time, making the difference unobvious. In this study, we assessed durability of ESWT by evaluating participants during a 12-week follow-up and the result indicated that ESWT could relieve pain and improve depression state. In another study (33), ESWT relieved pain for Morton's neuroma at a 12-week follow-up, which was similar to this study. In addition, durability could be assessed by more measurement methods, such as recurrence rate at the longer follow-up visit, which could be improved in future studies.

Interaction effects for baseline confounders, such as age, sex, BMI, invasive treatment, and PHN nerve segments were explored in this study, and no interaction effect for baseline confounders was observed. PHN duration was not added into this analysis because of some outliers. The result indicated that the age was correlated with the NRS and BPI scores. Hyperalgesia is more common in elderly population, as well as prolonged pain development and less effective medication, which might influence the quality of life (34). Furthermore, the result suggested that invasive treatment was related to PSQI scores. In some studies, pulse-modulated radio frequency affects brain physiology, which might explain the correlation between the invasive treatment and sleep quality (35).

In a systematic review, 20.7% of patients developed transient pain, swelling, petechiae, and other side effect after ESWT (36), which was related to high-dose ESWT, constant energy level, and radial shockwave therapy (36). In our study, ESWT was performed by a skilled therapist to ensure a low dose of energy (1–4 bar) gradually increasing according to patients' reaction, which might be the potential reason of no adverse reactions in this study. The result suggested that ESWT could be a non-invasive and safe treatment if under administration of low-dose, gradually progressively energy.

There are some limitations. Further studies with objective measurement and therapeutic mechanism are needed. Furthermore, a high proportion (31%) of patients were lost to follow-up, which could introduce potential bias. Some old patients had difficulty in telephone use and were lost to follow-up. Optimizing follow-up protocol and extending follow-up may be necessary in the future. In addition, the single-blind method may have the risk of bias. More double-blind and multicenter studies would be needed to confirm the result.

In conclusion, ESWT combined with conventional treatment could relieve pain and improve the psychological state of patients with PHN without serious adverse effects. Further randomized clinical studies are needed to confirm these results, so that ESWT could be used as a safe and effective complementary treatment for PHN.

## Data availability statement

The raw data supporting the conclusions of this article will be made available by the authors, without undue reservation.

## Ethics statement

The studies involving human participants were reviewed and approved by the Ethics Committee of West China Hospital, Sichuan University, Chengdu, China (No. 2019[814], date of approval: 30th December 2019). The patients/participants provided their written informed consent to participate in this study.

## Author contributions

LC designed this randomized controlled trial, collected the data, and wrote the article. PY and LY performed the statistical analysis, revised the manuscript, and provided final approval to the version of the study. AQ performed treatment in this study. TZ revised the manuscript and gave final approval to the version of the study. All authors discussed the results and commented on the manuscript.

## Funding

This study is supported by grant 2019HXFH069 from the 1·3·5 project for disciplines of excellence—Clinical Research Incubation Project, West China Hospital, Sichuan University, the grant 2018JY0105 from the Science and Technology Department of Sichuan Province, CYTD17-05 Innovation grant of the Chengdu Medical College and CYFY-GQ01 Startup foundation for Distinguished Scholars of the First Affiliated Hospital of Chengdu Medical College, and 1·3·5 project for disciplines of excellence (ZYJC21008), West China Hospital, Sichuan University (to TZ). These projects will not participate in any aspect of the trial, including design, data collection, analysis, or interpretation.

## Conflict of interest

The authors declare that the research was conducted in the absence of any commercial or financial relationships that could be construed as a potential conflict of interest.

## Publisher's note

All claims expressed in this article are solely those of the authors and do not necessarily represent those of their affiliated organizations, or those of the publisher, the editors and the reviewers. Any product that may be evaluated in this article, or claim that may be made by its manufacturer, is not guaranteed or endorsed by the publisher.
